# Pickleball- and Paddleball-Related Injuries in the Lower Extremity: Description, Treatment Options, and Return to Play

**DOI:** 10.7759/cureus.53954

**Published:** 2024-02-10

**Authors:** Olivia A Opara, Parker L Brush, Nicholas Pohl, Sebastian Fras, Daren Aita, Joshua Hornstein, Daniel Fletcher, Selene Parekh

**Affiliations:** 1 Department of Orthopedic Surgery, Rothman Orthopaedic Institute, Philadelphia, USA; 2 Division of Hand Surgery, Rothman Orthopaedic Institute, Philadelphia, USA; 3 Division of Sports Medicine, Rothman Orthopaedic Institute, Philadelphia, USA; 4 Division of Foot and Ankle Surgery, Rothman Orthopaedic Institute, Philadelphia, USA

**Keywords:** return to play, injury treatment, lower extremity, paddleball, pickleball

## Abstract

Background

Pickleball and paddleball are the fastest-growing sports in the United States. However, there are limited studies on the types of lower extremity injuries and treatment options in an outpatient clinic setting.

Hypothesis/purpose

This study reports the incidence rate, treatments, and return-to-play (RTP) outcomes for patients presenting to a single orthopedic outpatient center with pickleball- and paddleball-related lower extremity injuries.

Study design

This study is a retrospective case series, with level IV evidence.

Methods

A database search of our multispecialty electronic medical record (EMR) system from 2015 to 2023 identified 166 patients with outpatient pickleball- and paddleball-related lower extremity injuries. The retrospective data were reviewed for patient demographics, injury type, mechanism of injury, surgical or non-surgical treatment, and return-to-play recommendations.

Results

We observed that the majority of the patients with pickleball- and paddleball-related injuries in the lower extremities were over 60 years of age, with more males. Additionally, most injuries encountered were ankle sprain/strain from a twisting mechanism, which was treated non-surgically. Additionally, a significant number of patients suffered an Achilles tendon rupture (12.0%), which was treated surgically with an Achilles tendon repair (88.1%), accounting for the most common surgical treatment performed in this study. Of the 166 patients who were seen and treated, 68 (40.9%) returned to play, and 93 (56.3%) were lost to follow-up.

Conclusion

Most of these injuries were seen in the older population and caused by a sprain or strain due to sudden changes in direction, which were treated non-surgically. The most common surgical treatment was an Achilles tendon repair due to an Achilles tendon rupture. Although a relatively good number of patients were cleared to return to play, some patients were lost to follow-up. Meanwhile, some patients were advised to stop playing pickleball or paddleball due to the severity of their injuries. As this sport continues to rise in popularity and with the incidence rate of lower extremity injuries increasing over time, orthopedic surgeons should be aware of the types of injuries, treatment options, and outcomes, as well as ways to advise patients on prevention. Therefore, further research on the standard treatments and outcomes of pickleball- and paddleball-related injuries in the lower extremities is encouraged.

## Introduction

Pickleball and paddleball are two of the fastest-growing racket sports in the United States, with player numbers growing to 3.1 million in 2018, representing a 650% increase in membership since 2013 [1]. Pickleball is played on an indoor or outdoor court using a “ping-pong paddle”-type racket and a wiffle ball, mixing the rules of tennis and ping-pong. Paddleball is played indoors or outdoors without a net, with the ball bouncing off the wall, and the players are not allowed to have the ball bounce more than once on the floor [2]. The sport of pickleball and paddleball is mostly played among active adults as a recreational sport and low-impact exercise [3]. Additionally, it also serves as a motive for fitness, psychological well-being, competitiveness, and socialization [3-5].

Traumatic knee injuries, ankle injuries, and various muscle injuries have been associated with racquet sports such as tennis, racquetball, badminton, handball, and squash [6-8]. Although the games pickleball and paddleball are somewhat alike compared to other racquet sports, the style of play of the sport varies, leading to a diverse range of injuries sustained through various mechanisms during play. Pickleball and paddleball have gained popularity among active adults and are recognized as the fastest-growing sports since the COVID-19 pandemic among younger players between ages 13 and 34, owing to the social nature of the sport [9]. The diversity in age groups among pickleball and paddleball players has reshaped the number and types of injuries presented to outpatient orthopedic clinics in the United States.

The primary aim of this study is to analyze and describe pickleball- and paddleball-related lower extremity injuries that present in an orthopedic outpatient setting. The secondary aim is to describe the most common injury goal in addition to treatment options and management.

## Materials and methods

The Thomas Jefferson University Institutional Review Board (IRB) issued approval iRISID-2023-2490, and a waiver of informed consent per institutional protocol was obtained. After the IRB approval, a retrospective database search in our multispecialty internal electronic medical records (EMR) using the keywords “pickleball” and “paddleball” to create all pickleball- and paddleball-related lower extremity injuries was conducted. This included every patient who played pickleball as a recreational or professional sport. The data from 2015 to 2023 were collected and reviewed, identifying 365 patients with pickleball- and paddleball-related injuries. Patients over 18 years of age and with injuries obtained from playing pickleball or paddleball were included in the study. After a meticulous search, 166 patients met the inclusion criteria for pickleball- and paddleball-related lower extremity injuries. The patients were excluded if they had lower extremity injuries unrelated to pickleball or paddleball.

The data obtained during the chart review were selected for demographics, the anatomic location of the injury, the type of injury, the mechanism of injury, the timing between injury and first clinical assessment, and treatment interventions. Treatment was categorized as surgical or non-surgical treatment, surgical procedure, time to surgery from the initial visit, and return to play (RTP)/follow-up. The patients who were treated conservatively were also recorded. The data were thoroughly searched to ensure that pickleball- and paddleball-related injuries were identified.

Descriptive statistics was performed to calculate, describe, and summarize the collected research data, and analyses were performed using Excel version 16 (Microsoft® Corp., Redmond, WA).

## Results

**Figure 1:**
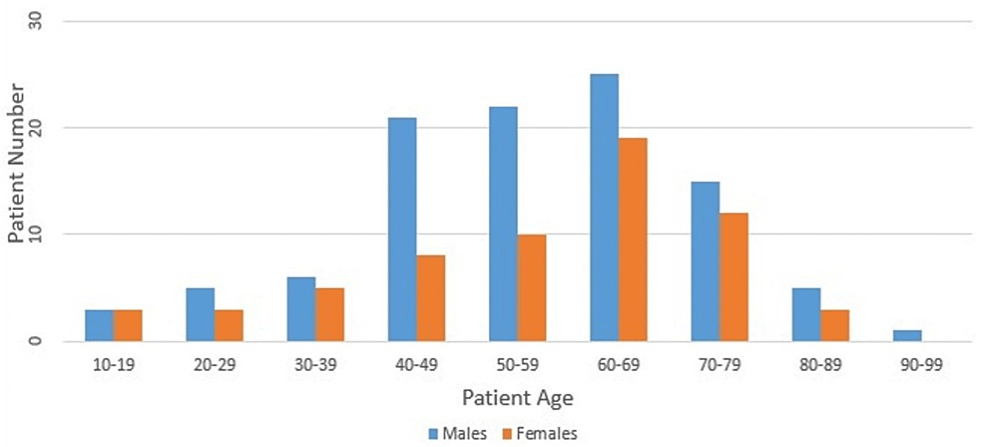
Age distribution based on sex

A total of 166 patients were identified, of which 93 (62%) were males and 73 (37.9%) were females. One hundred twenty-eight had pickleball-related injuries, and 38 had paddleball-related injuries. The patient demographics included in this cohort were 93 males (62%) and 73 females (37.9%). The patients’ age was between 60 and 69 years, with a mean of 63 years and a range of 15-94 years (Figure 1). The average time to initial visit was 38 days (range: 0-730 days) for both surgically and non-surgically treated patients. Of the 166 patients who presented with pickleball- and paddleball-related injuries, 42 (25.3%) were treated surgically and presented to the outpatient clinic at an average time of 48.4 days (range: 0-730 days) from injury to the initial visit. Additionally, the average time to surgery from the initial visit was 50.5 days (range: 0-510 days). On the other hand, the conservatively treated patients accounted for 127 (74.6%) of the cohort. The average time from injury to initial visit was 34.9 days (range: 0-365 days).

The categorization of pickleball- and paddleball-related injuries based on laterality, anatomic location, injury type, and the mechanism of injury is presented in Table 1. Ninety-two (56.1%) patients presented with right lower extremity injuries, while 70 (42.6%) presented with left lower extremity injuries. Four (2.41%) patients presented with bilateral injuries. The majority of injuries were associated with the foot and Achilles tendon, accounting for 56 (32.8%) and 55 (32.2%) of the total anatomic injuries. The most common injury presented to the outpatient clinic was a sprain injury of the ankle (36, 21.7%). Additionally, the most common mechanism of injury involved a sudden change in direction during sport.

**Table 1:** Pickleball- and paddleball-related injuries

Laterality (N=166 patients)	N (%)
Bilateral injuries	4 (2.41)
Left	70 (42.2)
Right	92 (55.4)
Injured anatomic location (N=171 patients, some with multiple injuries)	N (%)
Achilles	55 (32.2)
Ankle	38 (22.2)
Foot	56 (32.8)
Calf	3 (1.6)
Leg	9 (5.2)
Tibia	3 (1.8)
Fibula	2 (1.1)
Heel	5 (2.9)
Type of injury (N=166 patients)	N (%)
Ankle sprain/strain	36 (21.7)
Achilles tendon injuries	
Rupture	20 (12.1)
Bursitis	10 (6.0)
Tendinitis	14 (8.4)
Fractures	18 (10.8)
Contusion	3 (1.8)
Peroneal tendon injuries	
Rupture	1 (0.6)
Tendinitis	5 (3.0)
Flexor tendon rupture	12 (7.2)
Plantar injuries	
Fasciitis	11 (6.6)
Rupture	6 (3.6)
Fibromatosis	6 (3.6)
Others	24 (14.5)
Mechanism of injury (N=166 patients)	N (%)
Sudden change in direction	90 (54.2)
Twisting/rotational injury	26 (15.7)
Push off/jump/lunge	10 (6.02)
Fall/slip	8 (4.8)
Inversion	7 (4.2)
Unknown	25 (15.0)

Most patients treated surgically underwent an Achilles tendon repair due to full or partial tendon rupture (37, 88.1%). Conservative treatment included steroid injections (11, 4.2%), physical therapy (92, 35.7%), and orthotics/splint/brace (147, 57.1%), with seven patients (2.7%) who had multiple conservative treatment regimens included in their care. To monitor treatment progress, the patients were instructed to follow up for further evaluation and counsel for a possible return to play. Of the 166 surgically and non-surgically treated patients, 68 (40.9%) were cleared to return to play, and five (3%) were not cleared to return to play. They were instructed to refrain from pickleball or high-impact activities due to the tenosynovitis of the foot or posterior tibial tendonitis. However, most of the patients (93, 56%) treated were lost to follow-up, with no record indicating if they ever returned to play (Table 2).

**Table 2:** Treatments and return to play

Surgical treatment (N=42 patients)	N (%)
Achilles tendon repair	37 (88.1)
Ankle arthroscopy with peroneal tenodesis and the exploration of sural nerve	1 (2.4)
Open reduction and internal fixation ankle fracture	1 (2.4)
Plantar fascial release and gastrocnemius recession	1 (2.4)
Flexor tendon repair	1 (2.4)
Condylectomy of the fourth and fifth proximal phalanges of the left foot	1 (2.4)
Conservative treatment (N=257 patients; some patients had more than one treatment)	N (%)
Steroid injections	11 (4.2)
Physical therapy	92 (35.7)
Orthotics/brace/splint	147 (57.1)
Two or more treatments	7 (2.7)
Return to play/lost to follow-up (N=166 patients)	N (%)
Yes	68 (40.9)
No	5 (3.0)
Unknown/lost to follow-up	93 (56.0)

## Discussion

Given the limited number of research articles on pickleball and paddleball injuries in an outpatient setting, our study was conducted to highlight the injuries and treatment outcomes presented in an outpatient multispecialty orthopedic clinic. Our retrospective study identified 166 patients with lower extremity pickleball- and paddleball-related injuries over eight years, and the results were categorized based on types of injuries, types of treatments, and follow-up to provide a comprehensive understanding of treatment outcomes. Compared to other studies associated with racquetball-related injuries, there are not that many pickleball- and paddleball-related lower extremity injury cases [6-8,10]. Our findings suggest that the most common injuries in the lower extremities were ankle strain/sprain from sudden changes in movement when playing pickleball and paddleball. Many of these injuries were treated conservatively with orthotics/splints/braces, with Achilles tendon repair accounting for the most preferable surgical option.

A study by Kasper et al. identified 204 pickleball- and paddleball-related injuries in the upper extremities over eight years compared to our seven-year findings of 166 pickleball- and paddleball-related injuries in the lower extremities [10]. In contrast, Forrester identified a higher portion of lower extremity injuries presented to the emergency department (ED) than that of the upper extremities (32% and 25%, respectively) [11]. When interpreting our findings, we can infer that some lower extremity injuries were treated conservatively in the ED, hence the reason for fewer cases presenting to an outpatient orthopedic clinic.

Forrester also identified 300 patients with pickleball injuries in the lower extremities over five years (2013-2017) from a national database correlating to ED visits [11]. The author concluded that the most common lower extremity injuries identified in this study were sprains and fractures (28.7% and 27.7%, respectively). Although we obtained fewer cases of patients with lower extremity pickleball- and paddleball-related injuries, this supports our findings indicating that the most common lower extremity pickleball- and paddleball-related injuries were sprain or strain (21.6%), followed by fractures (10.8%). On the contrary, according to the study conducted by Kasper et al., the majority of the upper extremity injuries were fractures (39.7%) compared to strains (23%), suggesting that upper extremity injuries were higher than lower extremity injuries because upper extremity fractures were most likely to be followed in an outpatient clinic by an orthopedic physician for treatment compared to lower extremity sprains, which were more likely treated in the ED [10].

Additionally, a study conducted by Weiss et al. concluded that the most common diagnosis of pickleball- and paddleball-related injuries was a sprain or strain injury (33.2, 95% CI = 27.8%-39.5%), fractures (28.1, 95% CI = 24.3%-32.4%), and contusions (10.6, 95% CI = 8.0%-14.1%) [12]. The authors also identified that the majority of the patients diagnosed with pickleball and paddleball sprain and strain injuries were older males (odds ratio {OR} = 3.5; 95% CI = 2.2%-5.6%) compared to females (OR = 3.7; 95% CI = 2.3%-5.7%) who were most likely to suffer from a fracture. These findings correlated with our study that older males were mainly diagnosed with pickleball- and paddleball-related injuries. Most patients with pickleball injuries are over 60 years of age due to the low-impact nature of the sport and how easy it is to understand [3,10,12]. However, pickleball-related injuries in the younger population have become a concern due to the fast-growing nature of the sport with young adults over the recent years [9]. Continued research on this topic will provide healthcare professionals with the information to best advise patients on injury prevention and treatment outcomes. Greiner discussed the treatment of sprains and strains of the ankle and Achilles tendon, which includes limiting weight-bearing with crutches or immobilization with a brace [3]. This supports our findings that the majority of the ankle sprain and strain injuries were treated with orthotics/splints/brace to limit further injury and support mobilization and weight-bearing.

An article written by Quail, who played pickleball in the past and has knowledge of the common injuries obtained, encourages prevention, which includes specific stretching, warm-up exercises, proper footwear, ankle support braces, hydration, and taking frequent breaks [13].

In our study findings, the majority of pickleball- and paddleball-related injuries were due to sudden change in direction during play. Vitale and Liu advised players to learn the pickleball split to familiarize themselves with the “ready position” [14]. The authors also suggest that this position keeps the player’s feet and weight balanced to reduce the risk of ankle sprains, falls, or fractures. Due to the pickleball court size measuring 20 feet by 44 feet and the restricted area on the court that a player cannot step into, known as the kitchen, these two items restrict the areas in which the athletes move [14]. For this reason, athletes are encouraged to be aware of sudden stops, which require body control to avoid stepping into one’s own kitchen to drop a ball, as this can increase the risk of tendon tears and ankle sprains [12,14].

The findings from our study demonstrated that 86% of the patients were treated with non-surgical medications (nonsteroidal anti-inflammatory drugs or steroids), physical therapy, and splint/brace, while some patients required more than one conservative treatment modality. A few studies have also demonstrated that the most common lower extremity injuries seen in pickleball and paddleball are commonly treated conservatively [3,11-13]. The most common surgery performed was Achilles tendon repair (88.1%) from Achilles tendon rupture due to a sudden change in direction (55.2%) during play.

Our retrospective study recorded and analyzed data based on the type of injury, mechanism of injury, and treatment options, including follow-up and return to play. Finally, we analyzed patients’ follow-up and return to play and found that 40.9% were cleared to return to play after a successful treatment outcome, while 56% of the patients were lost to follow-up. Only 3% of the patients were advised against playing pickleball or paddleball or performing high-impact exercises.

Like most retrospective studies, we encountered some limitations. The cases were identified using the keywords “pickle,” “paddle,” “pickleball,” and “paddleball” in our EMR system. The specific keywords were not able to identify every patient chart related to the pickleball and paddleball sport, so therefore, there is a possibility that important data were missing. In the data search were patients who mentioned that they enjoyed playing pickleball or paddleball in the visit notes, but the injury was unrelated to either sport. If a patient did not report that the injury was due to a pickleball- or paddleball-related event, the injury was not captured in our data. Therefore, a wider range of search terms might have been more beneficial regarding results. Additionally, the number of pickleball and paddleball lower extremity injuries not seen in an outpatient orthopedic clinic setting is unknown. We were also unable to capture the time from surgery to RTP. Another limitation is the design of this study. This is a study that describes data collected over a period of time. No quantitative analysis was performed, and therefore, the reliability and generalizability of the findings are limited.

## Conclusions

Our study demonstrated that most of the patients with pickleball- and paddleball-related lower external injuries were over 60 years of age, with more males affected than females. Most of these injuries were ankle sprains or strains due to sudden changes in direction, which were treated non-surgically. The most common surgical treatment was an Achilles tendon repair due to an Achilles tendon rupture. Although many patients were cleared to return to play, some patients were lost to follow-up. A few patients were advised to stop playing pickleball or paddleball due to the severity of their injuries. As pickleball and paddleball sports continue to grow, orthopedic surgeons are encouraged to be mindful of major injury types and prevent and manage these sports-related injuries to ensure successful treatment outcomes.

## References

[1] PickleBall PickleBall (2024). USA pickleball. https://usapickleball.org/.

[2] Mackie B (2022). Paddleball vs pickleball - are they the same?. Published December 2.

[3] Greiner N (2019). Pickleball: injury considerations in an increasingly popular sport. Mo Med.

[4] Casper JM, Jeon JH (2018). Psychological connection to pickleball: assessing motives and participation in older adults. J Aging Phys Act.

[5] Ryu J, Yang H, Kim AC, Kim KM, Heo J (2018). Understanding pickleball as a new leisure pursuit among older adults. Educ Gerontol.

[6] Wörner EA, Safran MR (2021). Racquet sports: tennis, badminton, racquetball, squash. Specific sports-related injuries.

[7] Bouché RT (2010). Racquet sports: tennis, badminton, squash, racquetball, and handball. Athletic footwear and orthoses in sports medicine.

[8] Chard MD, Lachmann SM (1987). Racquet sports--patterns of injury presenting to a sports injury clinic. Br J Sports Med.

[9] Crisman E. Pickleball (2023). Pickleball: it’s not just for adults. Young players now the fastest-growing age bracket in the beloved sport. Chattanooga Times.

[10] Kasper AA, Gibbons JL, Abboudi J (2023). Pickleball- and paddleball-related injuries to the upper extremity. Cureus.

[11] Forrester MB (2020). Pickleball-related injuries treated in emergency departments. J Emerg Med.

[12] Weiss H, Dougherty J, DiMaggio C (2021). Non-fatal senior pickleball and tennis-related injuries treated in United States emergency departments, 2010-2019. Inj Epidemiol.

[13] Quail MT (2019). Caring for patients with pickleball injuries. Nursing.

[14] Vitale K, Liu S (2020). Pickleball: review and clinical recommendations for this fast-growing sport. Curr Sports Med Rep.

